# Supplementary material for: Influence of Osteopathic Manipulative Treatment on the Quality of Life and the Intensity of Lumbopelvic Pain in Pregnant Women in the Third Trimester: A Prospective Observational Study
Source: Healthcare (Basel). 2023 Sep 14;11(18):2538. doi: 10.3390/healthcare11182538 (PMC10530515; doi:10.3390/healthcare11182538)
Supplement: Supplementary file 1 [file healthcare-11-02538-s001.zip › healthcare-2541270-supplementary.pdf]

Supplementary Materials:

S1 - OMT protocol:

1- Upper thoracic opening- BLT – The patient is seated with the practitioner behind and hands leaning on the patient's scapular girdle. The practitioner positions the thumbs on either side of the C7 spinous process and the fifth fingers to spread the shoulder from the acromioclavicular joint, with the other fingers on the clavicles.

2- Stretching of the pleural dome ligaments – Patient seated on the edge of the bed, with the practitioner standing behind, arm on the side to be treated on the practitioner's leg, in 30º abduction and head in inclination/rotation opposed to the arm in abduction.

3- Relaxation and stretching of the lateral lumbocostal arch – Patient and practitioner sitting side by side. The thumb of the practitioner's caudal hand is situated below the patient's 12th rib. Initially, the patient's trunk is moved to the opposite position, then homolateral inclination when the thumb is pressed in the body's medial direction.

4- Thoracic diaphragm technique–patient in lateral decubitus, the practitioner is behind the patient's lower torso. The abdominal hand gently settles into the softer part of the belly, above the pregnant uterus. The abdominal hand uses the side of the index finger as the guiding contact rather than the fingertips. Once the abdominal hand has settled slightly under the costal margin, it rests there for a moment whilst the practitioner engages the lower ribcage with the other hand. With the hands now slightly overlapped, this relationship is maintained throughout the technique's rest and release the tension.

5- The technique for lumbar spinal muscles- The patient is in the lateral decubitus position, and the practitioner faces the patient. Place the two hands superimposed on each other in contact with the lumbar musculature. It will pull the muscle mass in all directions to release tension. Do it bilaterally.

6- BLT lumbosacral junction, pelvis and hip - The patient is seated with feet held by the practitioner and knees extended. the patient should not bend backwards. The practitioner then moves the legs towards ease, and the legs are held in that position. While the legs are being held in this position, the patient is asked to slowly rotate the trunk to the facilitated side until the sacrum begins to move. At that moment, the patient stops turning and remains in this position until the tensions are relaxed.

7- Elevation of the pelvic floor in lateral decubitus – the seated practitioner places the elongated fingers on the patient's pelvic floor, between the ischiatic tuberosity and the rectum. Gently enter their fingertips in the cephalic direction during the expiration phase.

8- Decompression of the atlantooccipital joint – the practitioner makes contact close to the occipital condyles, leading to anterior support, added to lateral and cephalic tension.

9- Treatment of the floor of the mouth- the practitioner places their fingertips in a medial position to the jawline and applies uniform pressure on both sides to balance the existing muscular tensions. Stop when your fingers perceive that the tissue has softened.

10- Alternating rotation of the temporal – Gentle compression on the mastoids to move them in opposite direction.

Table S2

The Brazilian version of the Quality-of-Life Questionnaire -SF-36

1-In general, would you say your health is:

Excellent	Very Good	Good	Bad	Very Bad
1	2	3	4	5

2- How would you rate your overall health now compared to a year ago?

Much better	A little better	About the same	A little worse	Much worse
1	2	3	4	5

3- The following items are about activities you could do during an average day. Due to your health, would you find it difficult to do these activities? In this case, when?

Activities	Yes, it is challenging.	Yes, it is a little tricky.	No, it is not difficult.
a) Rigorous activities require a lot of effort, such as running, lifting heavy objects, and participating in strenuous sports	1	2	3
b) Moderate activities include moving a table, vacuuming, playing ball, and sweeping the house.	1	2	3
c) Lifting or carrying groceries	1	2	3
d) Climb several flights of stairs	1	2	3
e) Climb a flight of stairs	1	2	3
f) Bending, kneeling, or bending over	1	2	3
g) Walk more than 1 kilometer	1	2	3
h) Walk several blocks	1	2	3
i) Walk one block	1	2	3
j) Bathing or getting dressed	1	2	3

4- During the last four weeks, have you had any of the following problems with your work or any regular activity due to your physical health?

	Yes	No
a) Do you reduce the amount of time you devote to your work or other activities?	1	2
b) Did you perform fewer tasks than you would like?	1	2
c) Was limited in his type of work or other activities.	1	2
d) Had difficulty doing your job or other activities (e.g., required extra effort)	1	2

5- During the past four weeks, have you had any of the following problems with your work or other regular daily activities due to any emotional problems (such as feeling depressed or anxious)?

	Yes	No
a) Do you reduce the amount of time you devote to your work or other activities?	1	2
b) Did you perform fewer tasks than you would like?	1	2
c) Did not carry out or do any of the activities as carefully as usual.	1	2

6- During the last four weeks, how did your physical health or emotional problems interfere with your usual social activities, with family, friends, or in groups?

Not at all	Slightly	Moderately	Quite a bit	Extremely
1	2	3	4	5

7- How much body pain did you have during the last four weeks?

None	Very mild	Mild	Moderate	Severe	Very severe

1	2	3	4	5	6

8- During the past four weeks, how much did pain interfere with your regular work (including work around the house)?

Not at all	A little bit	Moderately	Quite a bit	Extremely
1	2	3	4	5

9- These questions are about how you feel and how everything has been going for you during the last four weeks. For each question, please give one answer closest to how you feel over the past four weeks.

	All the time	Most of the time	A good part of the time	Some of the time	A small amount of the time	Never
a) How long have you been feeling full of vigor, will, and strength?	1	2	3	4	5	6
b) How long have you felt like a very nervous person?	1	2	3	4	5	6
c) How long have you felt so depressed that nothing could cheer you up?	1	2	3	4	5	6
d) How long have you been feeling calm or peaceful?	1	2	3	4	5	6
e) How long have you been feeling very energetic?	1	2	3	4	5	6
f) How long have you felt discouraged or downcast?	1	2	3	4	5	6
g) How long have you been feeling drained?	1	2	3	4	5	6
h) How long have you felt like a happy person?	1	2	3	4	5	6
i) How long have you been feeling tired?	1	2	3	4	5	6

10- During the last four weeks, how often did your physical health or emotional problems interfere with your social activities (like visiting friends, relatives, etc.)?

All the time	Most of the time	Some of the time	A small part of the time	None of the time

1	2	3	4	5

11- How true or false is each of the statements for you?

	Definitely true	Mostly true	Don't know	Mostly false	Definitely-false mind
a) I tend to obey a little more quickly than other people	1	2	3	4	5
b) I am as healthy as anyone I know	1	2	3	4	5
c) I think my health will get worse	1	2	3	4	5
d) My health is excellent	1	2	3	4	5

Figure S1: VISUAL ANALOG PAIN SCALE

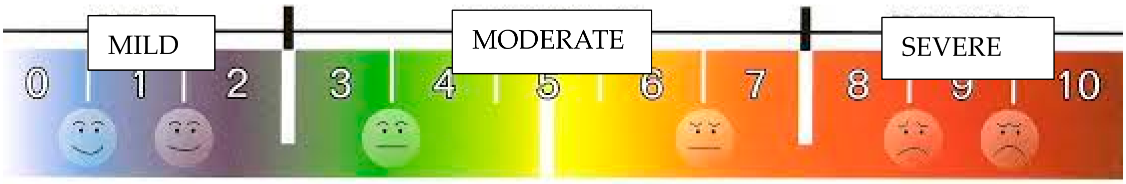

SOURCE: www.as.saude.ms.gov.br

Figure S2: Comparative bar graphs with mean low back and pelvic pain value before and after OMT in the sample.

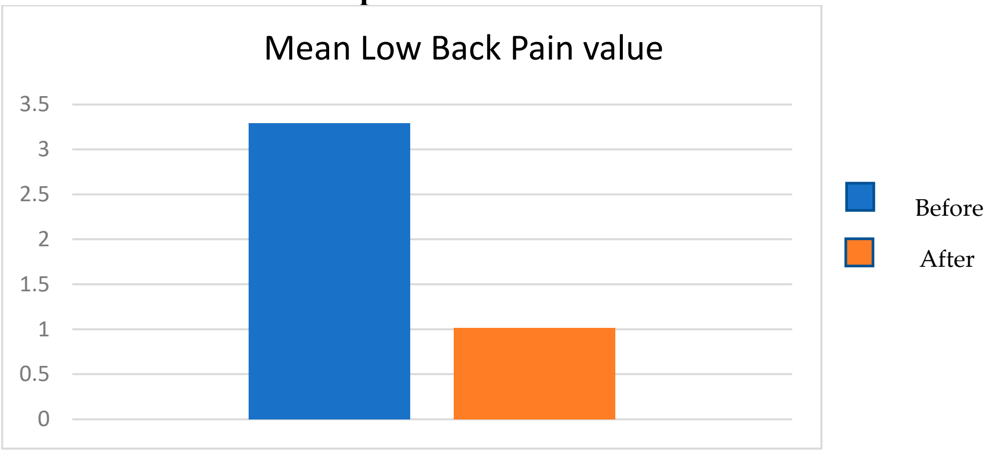

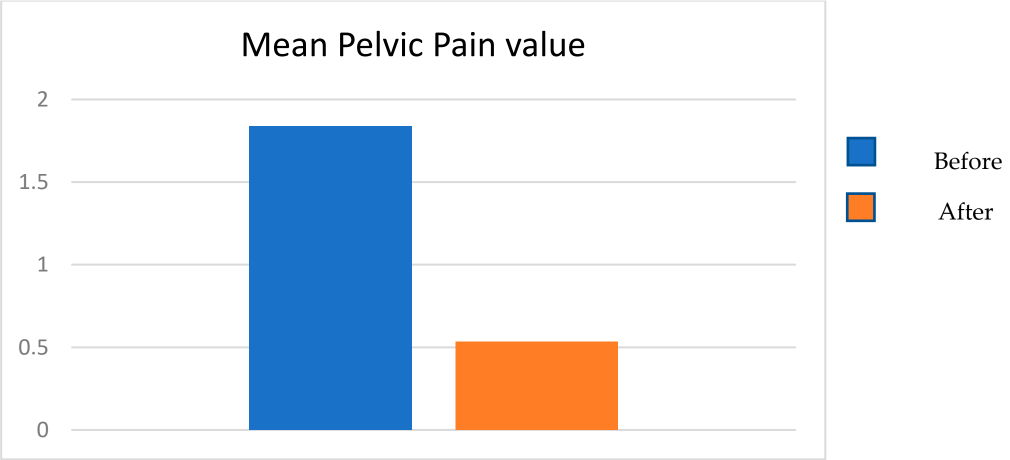

Variable	Before OMT				After OMT			
	**Overall**, N = 64	**≥ 4 OMT**, N = 27	**≤ 3 OMT**, N = 37	**p-value**	**Overall**, N = 64	**≥ 4 OMT**, N = 27	**≤ 3 OMT**, N = 37	**p-value**
**Low back pain -freq.**				<0.001*				**<0.001***
	37 (58%)	19 (70%)	15 (41%)		37(58%)	19 (70%)	15 (41%)	
**Low back pain - continuous**				0.442**				**0.800****
**Mean,SD**	3.3,3.4	3.1,3.3	3.6,3.5		1.0,1.8	0.9,1.7	1.1,2.0	
**Range**	0.0,10.0	0.0, 10.0	0.0, 10.0		0.0, 8.0	0.0, 8.0	0.0, 7.0	
**Pelvic pain-freq**				0.003*				**0.017***
	19 (30%)	11 (38%)	8 (20%)		18 (29%)	9 (35%)	8 (20%)	
**Pelvic-continuous**				0.084**				**0.030****
**Mean,SD**	1.9,3.0	1.5,2.6	2.9,3.8		0.6,1.3	0.3,0.8	1.1,2.0	
**Range**	0.0,10.0	0.0, 10.0	0.0, 9.0		0.0, 6.0	0.0, 3.0	0.0, 6.0	

*** Pearson's chi-squared test**

**** Two Sample t-test**

Low back pain affects 58%, and pelvic pain affects 30% of the population that participated in the study (table). In the bar graph, a decrease in the mean intensity of low back pain (3.3 ± 3.4 vs. 1.0 ± 1.8, p=0.800) and pelvic pain (1.9 ± 3.0 vs. 0 .6±1.3, p=0.03) of the sample can be observed. It is also noted that the intensity of both lumbar and pelvic pain decreases more in patients who had ≥four consultations, as well as the number of pregnant women who reported having pelvic pain (p=0.03).
